# Cortical layer multi-parameter analysis of neurovascular impairments in AD/ADRD rodent model with in vivo optical imaging

**DOI:** 10.1186/s40035-025-00530-4

**Published:** 2025-12-22

**Authors:** Hyomin Jeong, Jiaxiang Ren, Wensheng Cheng, Nora D. Volkow, Haibin Ling, Donghui Zhu, Congwu Du, Yingtian Pan

**Affiliations:** 1 Department of Biomedical Engineering, Stony Brook University, Stony Brook, NY 11794 USA; 2 Department of Computer Science, Stony Brook University, Stony Brook, NY 11794 USA; 3 National Institute on Alcohol Abuse and Alcoholism, National Institutes of Health, Bethesda, MD 20857 USA

**Keywords:** Apolipoprotein, Alzheimer’s disease, Dementia, Early diagnosis, Biomarkers, Neurovascular dysfunction, Cerebral vasculature, Optical imaging, Optical coherence tomography, Laser speckle contrast imaging, AI-based classifier

## Abstract

**Background:**

Neurovascular biomarkers have the potential to enhance early diagnosis of Alzheimer’s disease (AD) and AD-related dementias (ADRD), as cerebrovascular alterations often precede neurodegeneration. However, their clinical application remains challenging due to insufficient specificity, heterogeneity, and technical limitations.

**Methods:**

Here, we report that vessel- and cortical layer-specific parameters exhibit promising diagnostic sensitivity for neurovascular impairments in an AD/ADRD mouse model, apolipoprotein E (*APOE*) 4 knock-in (KI), compared to *APOE3*-KI at 12 months of age. Using two in vivo imaging modalities, 3D capillary-resolution optical Doppler tomography and laser speckle contrast imaging, we measured 36 morphological and functional vascular parameters and evaluated their diagnostic performance using a machine-learning Support Vector Machine classifier.

**Results:**

*APOE4* mice showed significant alterations including reduced venular and arterial cerebral blood flow velocities and diameters, increased vascular tortuosity, layer-dependent decreases in vascular density, and impaired cerebrovascular reactivity. Venule- and microcirculation-related parameters and dynamic vasoactivity to brain stimuli demonstrated high diagnostic accuracy (~ 90%).

**Conclusion:**

Together, these findings provide in vivo evidence for early, cortical layer-specific neurovascular dysfunction caused by *APOE4* that increases the susceptibility to dementia and highlight the potential of combining neurovascular biomarkers from optical imaging with AI-based classifier for identification of increased AD/ADRD risk.

**Supplementary Information:**

The online version contains supplementary material available at 10.1186/s40035-025-00530-4.

## Background

The E4 variant of apolipoprotein E (*APOE*), the most prominent genetic risk factor for Alzheimer’s disease (AD) [1, 2] and other forms of dementia [3, 4], dysregulates lipid metabolism in astrocytes [5] and amyloid-beta (Aβ) clearance [6] with increased neuroinflammation [7], which collectively exacerbate cognitive dysfunction. Moreover, breakdown of blood–brain barrier (BBB) is frequently observed in E4-carriers [8, 9] and in transgenic mice [10, 11], potentially triggering synaptic deficits seen in individuals with dementia [12]. Elucidating the pathological effects of E4 is particularly important given that approximately half of AD patients are projected to be E4-carriers [13, 14].

However, evidence on how the cerebrovasculature of E4-carriers differs from that of non-carriers is insufficient, despite the recent recognition that vascular impairment is one of the first pathological changes in dementia [15, 16]. Such difficulties stem from the complexity of the vasculature and limited imaging modalities to observe microvascular changes. Most prior studies utilized ex vivo immunohistochemistry (IHC) for assessing deeper brain morphology [17, 18]. However, this approach is prone to staining artifacts and loses 3D spatial information and physiological context of the vascular architecture. Clinically, functional magnetic resonance imaging (fMRI) [19] and positron emission tomography (PET) [20] allow noninvasive, in vivo measurement of region-specific vascular changes in the whole brain. Nonetheless, their low spatiotemporal resolutions limit their sensitivity for resolving individual cerebral vessels. Alternatively, optical methods such as two-photon microscopy (TPM) enable visualization of neocortical cerebrovasculature in vivo [21], but their limited field-of-view (FOV) hinders the assessment of heterogeneous cerebrovasculature in a single imaging session. Additional issues such as potential photobleaching and fluorescent marker artifacts may complicate cerebral hemodynamic measurements.

Another significant challenge in medical image analysis is human bias, which can influence data interpretation and introduce inconsistencies across studies, especially when differences between groups are subtle. A number of subjective decisions may be involved during manual processing, feature measurement, and diagnosis/classification. As the use of in vivo imaging systems continues to expand in studies of neurological disorders [22], there is a growing demand for efficient, automated analytical methods such as artificial intelligence (AI)-assisted approaches that can enhance precision and reproducibility.

Here, we present a comprehensive analysis of the pathological effects of the E4 allele on the cerebrovasculature in a layer- and vessel-specific manner in comparison to its neutral variant E3 in *APOE* knock-in (KI) models, using a machine-learning classifier, Support Vector Machine (SVM), based on the measurement of 36 vascular parameters. We used our custom Optical Coherence Tomography (OCT), which simultaneously images 3D ultrahigh-resolution cerebrovasculature (ultrahigh-resolution optical coherence angiography [µOCA]) and cerebral blood flow velocity (CBFv) at capillary resolution (ultrahigh-resolution optical Doppler tomography [µODT]) over a large FOV without the need for external markers [23, 24]. The captured images of the somatosensory cortex (SSC) of E4 and E3 mice were used to analyze various vascular parameters including CBFv, vessel diameter, and tortuosity of vessels on the pial surface. The microvascular network in deeper cortical regions was visualized in 3D to examine layer-dependent cortical changes including vascular density and bifurcation counts. Additionally, cerebrovascular reactivity (CVR) in the corresponding microvasculature was investigated by introducing either vasodilatory hypercapnia (5% CO_2_) or vasoconstrictive cocaine (1mg/kg, i.v.) to the mice while recording their temporal hemodynamic responses with Laser Speckle Contrast Imaging (LSCI) [25, 26]. These vascular parameters were used to train the SVM-based classifier, and its performance in distinguishing E4 from E3 was evaluated to identify the parameter(s) that could most accurately detect the differences between the two groups caused by E4-specific alterations. This AI-driven classification offers an advantage over conventional statistical methods by providing the predictive values of individual parameters for E3/E4 classification, enabling detection of E4-specific pathology in unlabeled samples for potential early-stage diagnosis. The animals were assessed at 12 months of age, which translates to 40 years of age in humans [27]. Given that neurodegenerative symptoms typically begin at age 65 [28] and *APOE4* carriers exhibit elevated biomarker levels as early as 55 [29], we selected this earlier time window to investigate whether cerebrovascular changes emerge prior to the established biomarker abnormalities in *APOE4*-KI mice. Given that continuous, adequate circulation of blood to the brain is essential in satisfying metabolic demands and preventing tissue ischemia along with neurological complications including cognitive decline, a full-scale, in vivo optical assessment of vascular changes in E4 mice concomitantly with AI-based classifier will provide novel insights into the role of E4 in vascular impairments that may serve as early-stage biomarkers relevant to increased AD risk.

## Methods

### Animal preparation

Female *APOE3*-KI (B6.Cg-Apoeem2(APOE*)Adiuj/J, Jackson Laboratory, Bar Harbor, ME, #029018) and *APOE4*-KI (B6(SJL)-Apoetm1.1(APOE*4)Adiuj/J, Jackson Lab, #027894) mice [30] (*n* = 5–7/group) at 12 months of age were examined in this study. A minimum sample size of 4.7 animals per group was estimated based on power analysis with 80% statistical power (1- β) at a type I error rate (α) of 0.05 [31]. This calculation was based on preliminary 3rd order artery CBFv measurements (0.675 ± 0.113 mm/s for E3 and 0.495 ± 0.081 mm/s for E4; *n* = 2 and 4–6 vessels/group). To ensure robustness, we included 5–7 animals per group. This sample size is also consistent with our previous in vivo imaging studies using cocaine-addiction models in rodents [32, 33], where similar sample sizes yielded robust, reproducible results.

Animals were housed in a temperature-, humidity-, and light-controlled environment where food and water were provided ad libitum. Prior to in vivo OCT and LSCI imaging, craniotomy of a 3 × 3 mm^2^ optical window was performed under 1.5% isoflurane anesthesia on the right SSC (A/P: + 0.5 mm, M/L: + 3.0 mm) following the steps previously reported by our lab [34]. Perioperative monitoring of body temperature and respiration rate (beats/min) was performed to maintain similar physiological conditions across different imaging sessions. The animals underwent post-operative care with 2.5 mg/kg Flunixin, an anti-inflammatory drug, for at least two days before imaging sessions to minimize residual surgical effects. Post-operative animal conditions and window clarity were monitored daily to ensure consistent imaging quality. No E3/E4-dependent differences associated with craniotomy were observed during the recovery period.

### In vivo imaging of multiple vascular parameters via OCT

Comprehensive profiling of multiple vascular parameters including quantitative CBFv, diameters, tortuosity of veins and arteries, and 3D layer-dependent microvascular density and bifurcation count, was achieved using our custom ultrahigh-resolution OCT (µOCT) [23, 24] and post-processing programs. The µOCT system is composed of µOCA and µODT that can simultaneously record angiograms and 3D vascular flow maps up to the capillary level. The system includes an ultra-broadband light source (λ = 1310 nm and λ_FWHM_ = 220 nm), a 2 × 2 broadband fiber-optic Michelson interferometer to achieve a high axial resolution (~ 2.5  µm), and a microscopic objective (f16mm/NA0.25) that focuses the collimated light onto the mouse cortex and collects the backscattered light to the detection fiber, in which a high-speed line scan InGaAs camera (2048-pixels, 145k-lines/s; GL2048, Sensors Unlimited) captures sequential OCT A-scans to achieve a transverse resolution of ~ 3 µm. The camera speeds were set at 6k A-lines/s for 3D µODT and 27k A-lines/s for µOCA in a relatively wide FOV of 2.0 × 1.9 × 1.4 cm^3^ to collect high-resolution images with a relatively short imaging time (~ 20 min). During the imaging, each animal was under 1.5% isoflurane in 100% oxygen, with head mounted onto a stereotaxic frame to minimize motion artifacts.

Post-processing of µODT images involved a custom Doppler flow reconstruction algorithm based on the phase subtraction method [35] and phase intensity method [23] to quantify a phase change caused by the Doppler flow of red blood cells between two consecutive A-scans into quantitative CBFvs and translate them to corresponding grayscale values to produce 3D CBFv maps. Three to 10 ROIs were selected along each vessel using ImageJ (National Institutes of Health, Bethesda, MD) to calculate its mean CBFv using the following Eq. [35]:

$$v = \frac{{\lambda }_{0}\Delta {\phi }_{max}}{4\pi nTcos{\theta }_{z}}$$

where λ_0_ refers to the central wavelength (1.31 µm), *n* is the refractive index (1.38), *T* is the time interval between two adjacent A-scans (≈0.167 ms), and *θ*_z_ is the angle between Doppler flow direction and the incident light. Veins and arteries were differentiated based on the flow direction of the measured Doppler flow because venular flow is directed upward to merge into larger veins, while arterial flow descends into smaller branches to facilitate cellular perfusion. These vessel types were further categorized into 1st-, 2nd-, and 3rd-order branches based on vascular morphology: 1st-order vessels are mainstream pial vessels; diving vessels, arterioles, and venules that bifurcated from the 1st-order vessels were defined as 2- and 3rd-order vessels consecutively. Veins were analyzed only up to 2nd-order because veins typically bifurcate less due to their converging nature to return to the heart rather than diverging like arteries to spread the blood flow.

Vessel diameters were measured by manually selecting pixel lengths of the same vessels quantified for CBFv in ImageJ and then converting these measurements to actual lengths. Similarly, multiple measurements nearby were taken on the same vessel and averaged to yield a representative diameter for each vessel, and 15–20 vessels per vessel type on average were reported in each group (*n* = 6–7 mice/group).

To quantify vessel tortuosity, a node (i.e., bifurcation/branching point) and its corresponding vessel endpoint were identified, and a direct length between these two points was measured to compare with an actual length of the vessel. The tortuosity index was defined as the ratio of the actual path length to the direct path length between two vessel nodes [36], where a value of 1 means a straight branch and values greater than 1 indicate increased tortuosity.

The layer-dependent microvascular analysis consisted of four major steps: (1) plane alignment, (2) vessel segmentation, (3) vessel skeletonization, and (4) vessel morphometric analysis. The first “plane alignment” involved tilting the ODT images to align the brain surface to a horizontal plane. This process was necessary because the OCT imaging setup inherently prevents the cranial glass reflections from being positioned perfectly perpendicular to the incident laser beam, which could otherwise saturate A-scans and compromise image quality. To correct this tilted plane, the plane of the glass window was identified, and the ODT images were adjusted entirely to align with the horizontal plane. This alignment enabled the precise division of horizontal layers at equal depths. The second step, “vessel segmentation,” was performed based on the Frangi algorithm [37], which automatically calculates the eigenvalues of the Hessian matrix. Its results were then used to computationally assess the similarity of an image region to vessels, which were successfully distinguished from the background in 3D. Then, morphology operations such as dilation and erosion were applied to denoise the images. The resulting image stacks were in a 3D binary of vessels versus the background. In the third step, “vessel skeletonization,” the binary vascular images were skeletonized in 3D based on the 3D parallel thinning algorithm [38]. In essence, this algorithm repeatedly scans the images and removes pixels at each iteration until the image remains unchanged. In each iteration, candidate pixels for removal are created first, and then they are rechecked sequentially to better maintain the connectivity of the image. The 3D vessels were then transformed into 3D skeletons with one-voxel thickness after this operation. Based on the 3D binary vascular and skeletonized images created, we computed quantitative parameters in different layers (L1-5, 100 μm each from the surface), including i) vessel density (vessel volume divided by layer volume), ii) skeleton density (skeleton volume divided by layer volume), and iii) bifurcation count (the total number of bifurcation nodes within a layer). The bifurcation count was quantified by identifying a vessel graph with nodes and edges in 3D and counting the vessel segments with three degrees of nodes as bifurcated branches.

### In vivo assessment of vasoactive CVR via LSCI

The functional ability of the brain to respond to vasoactive stimuli, known as CVR, was assessed using LSCI. This technique estimates blood perfusion, i.e., cerebral blood flow (CBF), with high spatiotemporal resolution by retrieving dynamic speckle patterns generated by moving red blood cells. Our custom LSCI contains a laser diode at 830 nm (DL8142-201–830, Thorlabs, Newton, NJ) coupled to a single-mode fiber that is illuminated over the cranial window in a 10-ms exposure. A zoom microscope (AZ100, Nikon, Tokyo, Japan), 2 × Plan APO objective, and an sCMOS camera (Zyla 5.5, Andor, Belfast, United Kingdom) were used to capture varying perfusion flows in a wide FOV (e.g. ~ 4 × 4 mm^2^) at 1 fps. The resulting raw LSCI images usually exhibit a high noise profile from light scattering and heterogeneity of tissue perfusion, and thus additional post-processing was performed to enhance the signal-to-noise ratio. We used a spatial-domain LSCI reconstruction algorithm reported previously [39], in which 5 by 5 adjacent pixels were binned within each frame to enhance sensitivity and spatial resolution. Five ROIs per animal were selected within the microvasculature area, and their CBF responses against vasoactive stimuli were quantified as relative percentage changes (ΔCBF) over the baseline (*t* = 0–5 min) before the stimuli. In the case of vasodilatory stimulus, mild hypercapnia was induced by changing the inhalation gas from 100% O_2_ to 5% CO_2_:95% O_2_ as it is known to induce vasodilation. On the other hand, vasoconstriction was triggered by acute (< 15 s) intravenous administration of cocaine (1 mg/kg) into the tail vein, as cocaine is known to induce global vasoconstriction in rodent brains [40–42].

### SVM-based classification for evaluating vascular parameters

To perform a systematic assessment of multivariant (i.e., 36) vascular parameters in detecting E4-specific neurovascular impairment, we implemented an SVM-based classifier [43] with the Radial Basis Function (RBF) kernel [44]. In essence, SVM identifies an optimal hyperplane that best separates the two groups based on supervised machine learning, and RBF allows non-linear relationships to be evaluated by mapping the data into a higher-dimensional space. For each vascular parameter, bootstrap resampling was performed to estimate E3 vs E4 classification accuracy and its confidence intervals. Bootstrap samples were generated by sampling with replacement from the original dataset. Specifically, for a group size of *n* (e.g., 12), *n* observations were sampled randomly with replacement to create a group of *n* bootstrap replicates while allowing individual observations to be selected multiple times. Then, leave-one-out cross-validation was applied on this set of replicates to compute the average classification accuracy for *n* number of trials. For example, when evaluating the parameter “skeletal density_L1”, the training–testing process was repeated 12 times (corresponding to a total of 12 animals in the two groups combined). In each trial, one of the animals was set aside as the test set while the model was trained on the remaining 11 animals. The trained SVM then attempted to classify the test animal in a blind manner. If the classification was correct, the trial received a score of 100% (1); if incorrect, it received 0% (0). This whole procedure was repeated 1000 times per parameter to yield the mean classification accuracy for each metric. The parameters with the highest classification accuracy were considered the most effective in distinguishing between the two groups, allowing us to identify vascular markers that may be useful for early detection of E4-specific neurovascular deficits.

### Statistics

Data are presented as mean ± standard error of the mean (SEM). A two-tailed Student’s *t*-test with the Shapiro–Wilk normality test was performed with the SigmaStat software (Systat Software Inc.) to compare the E3 and E4 groups. The significance level was set at *P* < 0.05 for all analyses. Differences in systemic physiological parameters (body temperature and respiration rate) were assessed using two-way repeated measures ANOVAs with Group (E3 vs E4) and Time (*t* = 1–35 min) as factors.

## Results

### Resting-state hypoperfusion and vasoconstriction in veins and arteries of E4 mice across branching orders

**Fig. 1:**
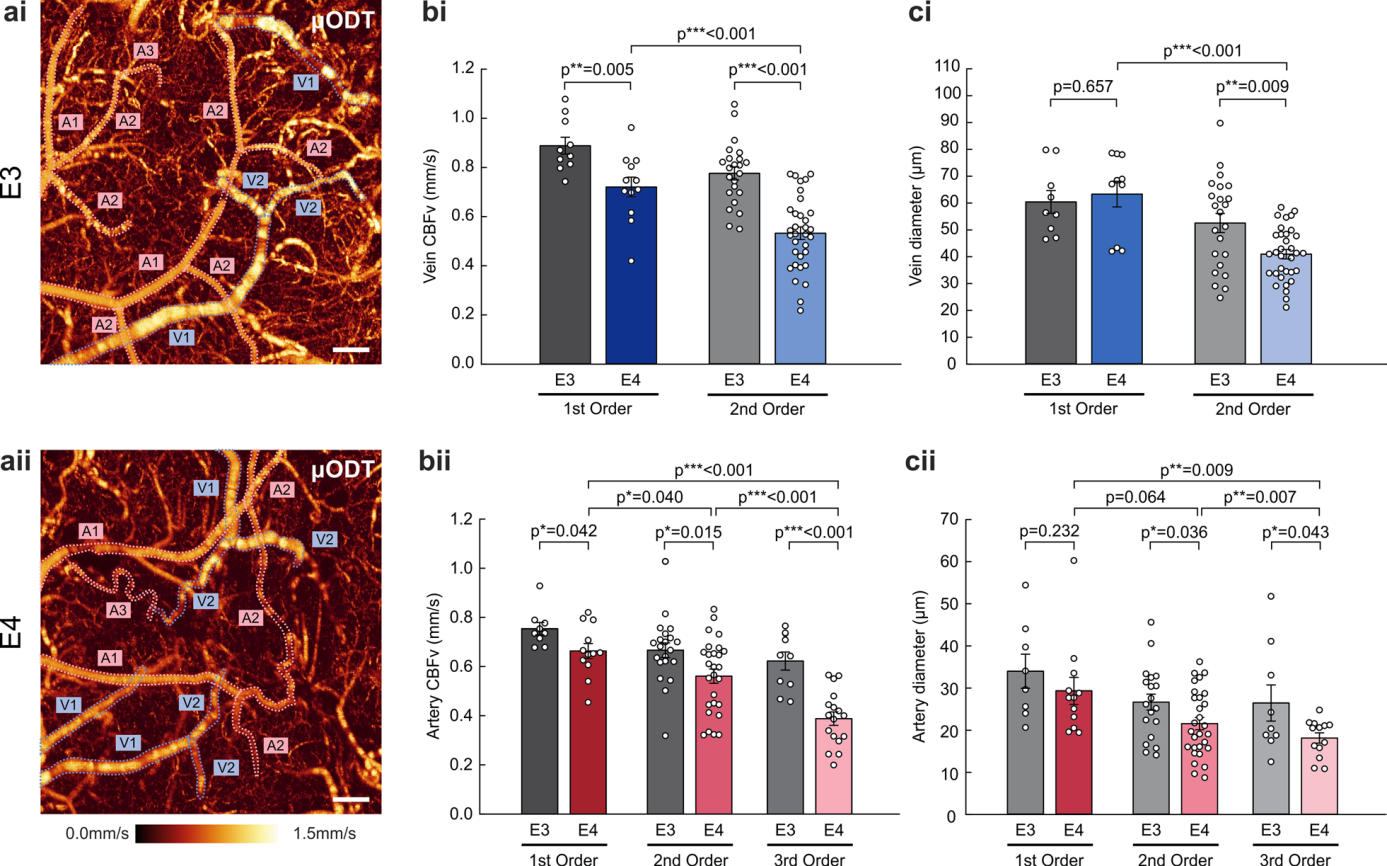
Reduced cerebral blood flow velocity and diameter of veins and arteries in the cortex of E4 mice. **a** μODT images showing CBFv rates of pial veins (V) and arteries (A) in the SSC of E3 (**i**, *n* = 6) and E4 (**ii**, *n* = 6). A 1st-order vessel (V1 or A1) indicates a large, pial vessel; a 2nd-order vessel (V2 or A2) refers to a secondary branch that bifurcates from the main vessel; and a 3rd-order vessel (A3) is a smaller branch that bifurcates from the 2nd-order vessel, which includes diving vessels and arterioles/venules. Scale bar: 200 µm. **b** CBFv (mm/s, mean ± SEM) of pial veins (**i**) and arteries (**ii**) in E3 (gray bars) and E4 (colored bars). The corresponding CBFv rates in E4 were significantly lower than in E3 in all vessel types and orders. **c** Flow diameters ϕ (µm, mean ± SEM) of pial veins (**i**) and arteries (**ii**) in E3 (gray bars) and E4 (colored bars). Higher-order (i.e., smaller) veins and arteries showed significant reductions in diameter in E4 vs E3 mice

We investigated the effect of E4 on resting-state CBFv and diameters of veins and arteries. The CBFv maps generated from one of the functional variants of our OCT, µODT, revealed that both venous and arterial flows of E4 **(**Fig. 1aii) were significantly reduced compared to those of E3 (Fig. 1ai), as visualized by the darkened color tone (i.e. lower flow) of the E4 image. To compare the two groups (*n* = 6–7/group) quantitatively, the vessels were divided into different orders: large, pial vessels as 1st-order vessels, and smaller vessels that bifurcated from 1st-order vessels consecutively as 2nd- and 3rd-order vessels, which consist of diving vessels, arterioles, and venules. The results showed significant CBFv reductions (*P* = 0.005 and *P* < 0.001) in both 1st- and 2nd-order veins of E4 (0.72 ± 0.04 and 0.53 ± 0.03 mm/s) compared to E3 (0.89 ± 0.03 and 0.78 ± 0.03 mm/s), respectively (Fig. 1bi). The percentage of decrease between the two groups was larger in 2nd-order (38%) than in 1st-order (21%) veins, suggesting that bifurcated veins were more severely affected by E4. Similar CBFv reductions of all-order arteries in E4 vs E3 were observed (Fig. 1bii). The 1st-order mean arterial CBFv rates were 0.66 ± 0.03 mm/s for E4 vs 0.75 ± 0.03 mm/s for E3 (*P* = 0.042). Those of bifurcated 2nd-order arteries were 0.56 ± 0.03 mm/s for E4 vs 0.67 ± 0.03 mm/s for E3 (*P* = 0.015) and of 3rd-order arteries were 0.39 ± 0.03 mm/s for E4 vs 0.62 ± 0.03 mm/s for E3 (*P* < 0.001), respectively. As with veins, the percentage CBFv decreases between E4 and E3 mice were 13%, 18%, and 47% for the 1st-, 2nd-, and 3rd-order arteries, respectively, with the 3rd-order arteries showing the most pronounced decline. Additionally, arteries in E4 exhibited a progressive decrease in CBFv across branching orders, as indicated by significant differences between 1st and 2nd (*P* = 0.004), 1st and 3rd (*P* < 0.001), and 2nd and 3rd (*P* < 0.001), whereas the differences between 2nd- and 3rd-order vessels were not significant in E3 (*P* = 0.381).

The mean diameter of 2nd-order veins was significantly smaller in E4 (40.92 ± 1.73 µm) than in E3 (52.57 ± 3.54 µm, *P* = 0.009), whereas no difference was found for 1st-order veins (63.34 ± 4.79 µm for E4 vs 60.42 ± 4.24 µm for E3, *P* = 0.657) (Fig. 1ci). Interestingly, there was a marked diameter reduction between 1st- and 2nd-venous flows of E4 (*P* < 0.001) while no significant change was detected in different orders of E3 (*P* = 0.22), which may suggest defective angiogenesis/bifurcation in E4 mice. As for arterial flows (Fig. 1cii), the bifurcated vessels exhibited smaller diameters in E4 than in E3 for 2nd-order (21.55 ± 1.46 µm vs 26.64 ± 1.90 µm, *P* = 0.036) and 3rd-order (18.13 ± 1.23 µm vs 26.45 ± 4.30 µm, *P* = 0.043). The 1st-order arteries did not exhibit any significant difference between E4 (14.66 ± 1.61 µm) and E3 (17.00 ± 2.03 µm, *P* = 0.232). Likewise, decreases from 1st to 3rd (*P* = 0.009) and from 2nd to 3rd (*P* = 0.007) were observed in E4, whereas no such order differences in arteries were observed in E3 (*P* = 0.072 for 1st vs 2nd, *P* = 0.224 for 1st vs 3rd, and *P* = 0.961 for 2nd vs 3rd). Altogether, these results showed significant vasoconstriction in both veins and arteries, particularly in higher-order branches of E4 vs E3 mice, which may explain the cause of hypoperfusion and subsequent damage to the neurovascular system in E4 mice.

### Increased venous and arterial tortuosity in the cortex of E4 compared to E3 mice

**Fig. 2:**
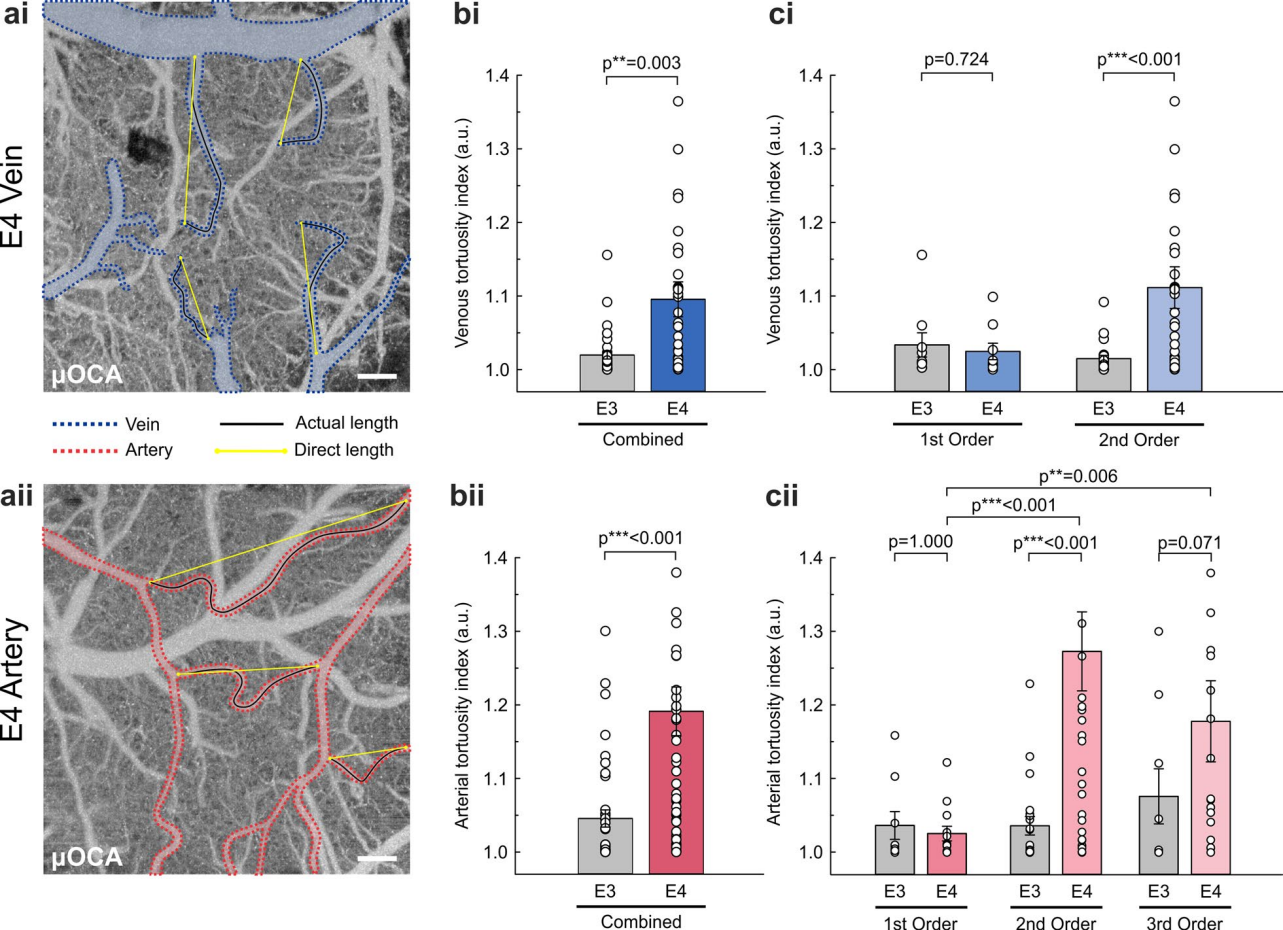
Wide-view evidence of tortuous vessels in the cortex of E4 vs E3 mice. **a** μOCA images of tortuous vessels observed in pial veins (**i**) and arteries (**ii**) of E4. Yellow straight lines connect the starting-to-ending points of a vessel branch to show the direct lengths; black curved lines follow the actual paths (lengths) of the vessels. The ratio between the actual and direct lengths was calculated to estimate the tortuosity index (a.u.). Scale bars: 200 µm. **b** Tortuosity indices (mean ± SEM) of pial veins (**i**) and arteries (**ii**) in E3 (gray bar) and E4 (colored bar). The cortical vessels in E4 are more tortuous than those in E3 for venous (*P* = 0.003) and arterial (*P* < 0.001) vessels. **c** Tortuosity indices (mean ± SEM) in pial veins (**i**) and arteries (**ii**) of E3 (gray) and E4 (colored) across branching orders. Higher-order vessels (i.e. venules and arterioles) were more tortuous in E4 while the level of tortuosity did not significantly differ over orders of vessels in the E3 brain

To characterize vascular deformities in E4 vs E3 mice and to demonstrate the capability of µOCA to visualize tortuous vessels over a large FOV, we quantified tortuosity index (a.u.) in cortical vessels of E3 and E4 mice (Fig. 2). Figure 2a shows examples of highly tortuous veins (i) and arteries (ii) observed in E4 mice. In the case of venules, C-shaped curves with an abrupt “kinking” towards the end were commonly observed in E4, and the venous tortuosity indices were significantly higher (*P* = 0.003) in E4 (1 .095 ± 0.024; *n* = 7, 49 vessels) than in E3 (1.020 ± 0.005; *n* = 6, 34 vessels) (Fig. 2bi). We further analyzed the vessel tortuosity based on branching hierarchy and showed that the 2nd-order venules in E4 (1.111 ± 0.028) were more tortuous than in E3 (1.015 ± 0.004, *P* < 0.001) (Fig. 2ci). The 1st-order veins did not differ between E4 and E3 mice (1.034 ± 0.016 vs 1.025 ± 0.011, *P* = 0.724), indicating that smaller, bifurcated vessels are more likely to be affected in E4. Similarly, the arterioles in E4 mice often showed S-shaped curves or coiling (Fig. 2aii), with higher tortuosity indices (1.19 ± 0.033, *n* = 7, 59 vessels) than in E3 (1.046 ± 0.012, *n* = 6, 39 vessels; *P* < 0.001) (Fig. 2bii). Figure 2cii shows that both 2nd-order arterioles (1.273 ± 0.054 vs 1.036 ± 0.013, *P* < 0.001) and 3rd-order arterioles (1.178 ± 0.056 vs 1.076 ± 0.037, *P* = 0.071) in E4 were more tortuous than those in E3, but the difference was not statistically significant as the tortuosity in E3 was high as well. In contrast, the 1st-order arteries were mostly smooth in both E3 and E4 mice (1.037 ± 0.019 vs 1.026 ± 0.010, *P* = 1.000). Overall, these results document morphological abnormalities in cortical veins and arteries of E4.

### Layer-dependent microvascular impairment in E4 as assessed by 3D cerebrovascular flow map

**Fig. 3:**
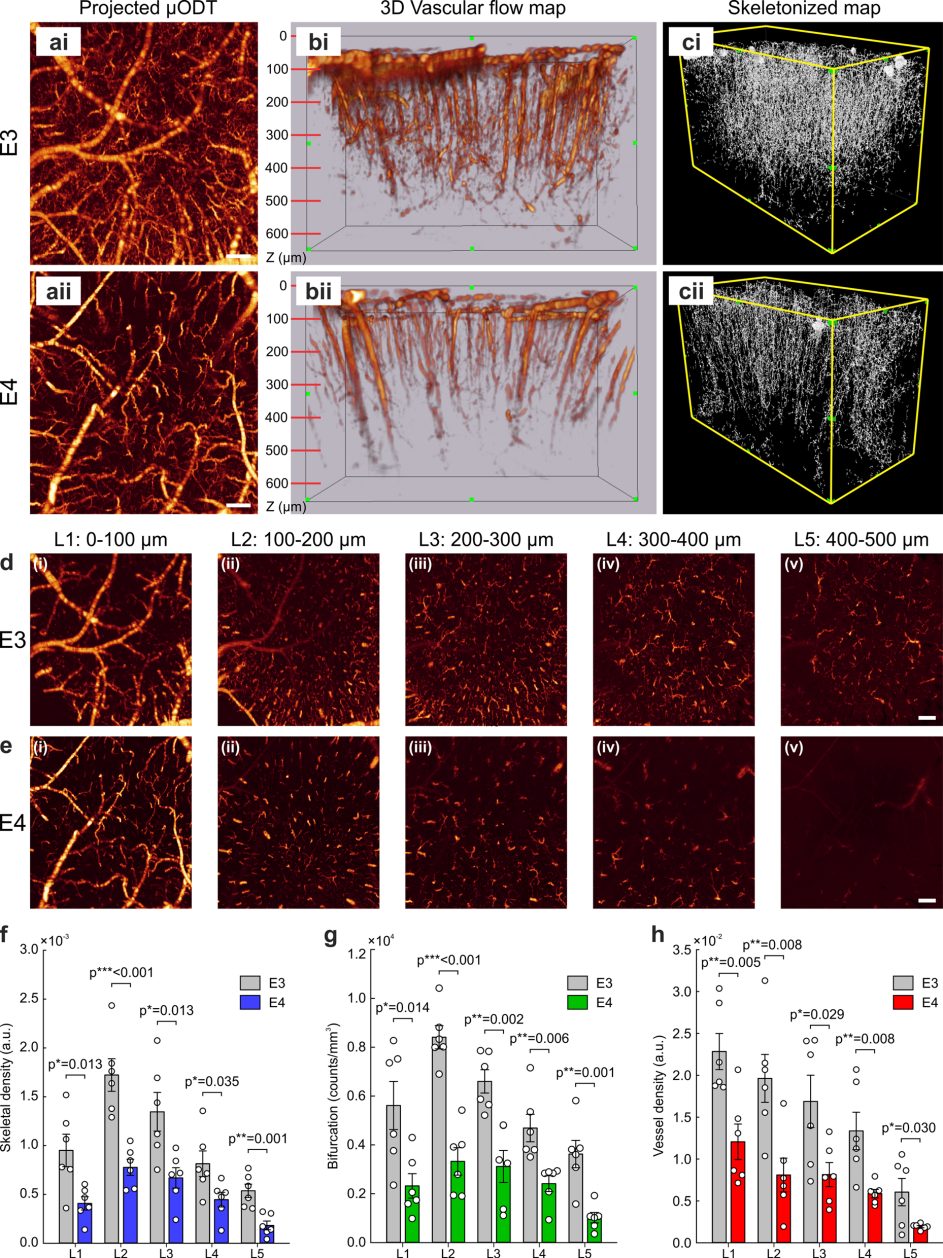
Layer-specific analysis of microvascular impairment in vivo in the cortex of E4 vs E3 mice. **a** Maximum-intensity-projection (MIP) μODT images of the cerebral cortex in E3 (**i**) and E4 (**ii**), showing noticeably reduced CBFv networks (e.g., capillary flows) in E4. Scale bar: 200 µm. **b** 3D μODT images (2 × 1.9 × 0.65 mm^3^) of the corresponding CBFv networks to 650 µm of depth in E3 (**i**) and E4 (**ii**) mice. While abundant microvessels were bifurcated from diving vessels and interconnected in E3, microvascular flow connectivity was visibly decreased in E4. **c** Skeletonized vasculature map of the corresponding cortices in E3 (**i**) and E4 (**ii**) mice. Automated identification of vasculature was followed by skeletonization to illustrate 3D vascular morphology and quantify skeletal density and bifurcation. **d, e** MIP images in different cortical layers (L1–L5) to illustrate CBFv distribution of the corresponding E3 and E4 mice. The CBFv networks were densely dispersed in all layers of E3, whereas those in E4 were sparsely distributed and their flow rates were weaker and barely detectable in the deeper cortex. L1: 0–100 µm, L2: 100–200 µm, L3: 200–300 µm, L4: 300–400 µm, L5: 400–500 µm. Scale bars: 200 µm. **f** Layer-dependent vascular skeletal density (a.u., mean ± SEM) in E3 and E4 mice (*n* = 6/group). Skeletal densities of E4 were significantly reduced in all layers compared to those of E3, especially in L2. **g** Layer-dependent bifurcation index (counts/mm^3^, mean ± SEM) in E3 and E4 mice. E4 showed reduced bifurcation indices, i.e., impaired vessel connectivity in all layers, especially in L2. **h** Layer-dependent vessel density (a.u., mean ± SEM) in E3 and E4. Vessel densities were significantly lower in E4 than in E3 mice in all layers

We further investigated the microvascular CBFv distribution within penetrating vessels and capillary networks in deeper cortical layers via quantitative vascular parameters from 3D µODT images (Fig. 3). Figure 3a presents maximum-intensity-projection (MIP) µODT images (2.0 × 1.9 mm^2^) of E3 (i) and E4 (ii). Although MIP is often shown to highlight global differences, e.g., the decrease in vessel density in E4 compared to E3 mice, it is unable to reveal depth-specific 3D features. However, based on the high sensitivity of our µODT and automated denoising and vessel connectivity, we present a side-3D-view of CBFv networks (Fig. 3b), which shows pial and penetrating vessels, venules, arterioles, and capillaries identified to measure the parameters described previously, and the skeletonized maps (Fig. 3c) from the brain surface to 650 µm of depth in E3 vs E4 mice. While abundant microflows were bifurcated from the penetrating cerebral flows and extended to deeper cortical layers in E3 (Fig. 3bi, ci), microvascular connectivity was largely reduced, and the microcirculatory flows were sparsely distributed in deeper layers of E4 (Fig. 3bii, cii). We then divided the cortex into five layers, L1 (0–100 μm), L2 (100–200 μm), L3 (200–300 μm), L4 (300–400 μm), and L5 (400–500 μm), to quantitatively assess layer-dependent changes in the microvascular networks and gain insight into their correlation to layer-specific neuronal populations (e.g., pyramidal neurons in L2/3 [45] and stellate neurons in L4 [46]). Figure 3d and e illustrate the corresponding MIPs of the CBFv networks at each layer for E3 and E4, respectively. The CBFv networks were densely dispersed in all layers of E3, whereas those in E4 were sparsely distributed with flows barely detectable in the deeper cortex.

The vascular skeletal densities were significantly reduced in all cortical layers of E4 vs E3 mice (*P*_L1_ = 0.013, *P*_L2_ < 0.001, *P*_L3_ = 0.013, *P*_L4_ = 0.035, and *P*_L5_ = 0.001) (Fig. 3f). The mean skeletal densities for E3 were 0.95 ± 0.17 (L1), 1.72 ± 0.17 (L2), 1.35 ± 0.20 (L3), 0.814 ± 0.13 (L4), and 5.37 ± 0.07 (L5) × 10^–3^ a.u.. The counterparts for E4 were 0.41 ± 0.07 (L1), 0.77 ± 0.08 (L2), 0.67 ± 0.11 (L3), 0.45 ± 0.08 (L4), and 0.18 ± 0.05 (L5) × 10^–3^ a.u.. Importantly, L2 of E4 mice showed the most significant reduction in skeletal density compared to E3.

To further assess angiogenic capability, a bifurcation index (counts/mm^3^) was quantified. The bifurcation counts were substantially decreased in E4 across all cortical layers (*P*_L1_ = 0.014, *P*_L2_ < 0.001, *P*_L3_ = 0.002, *P*_L4_ = 0.006, and *P*_L5_ = 0.001) (Fig. 3g), indicating that angiogenesis was reduced globally in E4 compared to E3. The mean bifurcation indices for E3 were 0.56 ± 0.10 (L1), 0.84 ± 0.05 (L2), 0.66 ± 0.05 (L3), 0.47 ± 0.06 (L4), and 0.36 ± 0.06 (L5) × 10^4^ counts/mm^3^, whereas those for E4 were 0.53 ± 0.06 (L1), 0.33 ± 0.06 (L2), 0.29 ± 0.06 (L3), 0.25 ± 0.03 (L4), and 0.12 ± 0.03 (L5) × 10^4^ counts/mm^3^. Consistent with skeletal density analysis, the largest reduction in bifurcation counts of E4 was observed in L2.

In addition, we examined another critical parameter, vessel density, based on µODT images with vasculature masks segmented in 3D (Fig. 3h). While skeletal density reflects the overall distribution of vasculature by reducing vessels to single-voxel skeletons through automated thinning, vessel density incorporates vessel volume, thereby providing an indirect measure of the total perfusion volume and distribution. Vessel densities (a.u.) showed significant reductions in E4 compared to E3 mice in all layers (*P*_L1_ = 0.005, *P*_L2_ = 0.008, *P*_L3_ = 0.029, *P*_L4_ = 0.008, and *P*_L5_ = 0.030). In E3, the mean vessel densities were 2.28 ± 0.22 (L1), 1.96 ± 0.29 (L2), 1.69 ± 0.31 (L3), 1.34 ± 0.22 (L4), and 0.61 ± 0.16 (L5) × 10^–2^ a.u., whereas those in E4 were 1.12 ± 0.21 (L1), 0.81 ± 0.20 (L2), 0.81 ± 0.14 (L3), 0.59 ± 0.05 (L4), and 0.19 ± 0.01(L5) × 10^–2^ a.u.. These global reductions in vessel densities imply that the vascular architecture for cerebral perfusion was significantly impaired in the cortex of E4 compared to E3. Also, the largest reductions were observed in L1 and L2. Taken together, these results reveal that vascularized areas for cerebral perfusion and angiogenesis are significantly diminished in the cortex of E4 mice, especially in L2 (100–200 μm from the pial surface).

### Dysfunctional CVR to both vasodilatory and vasoconstrictive stimuli

**Fig. 4:**
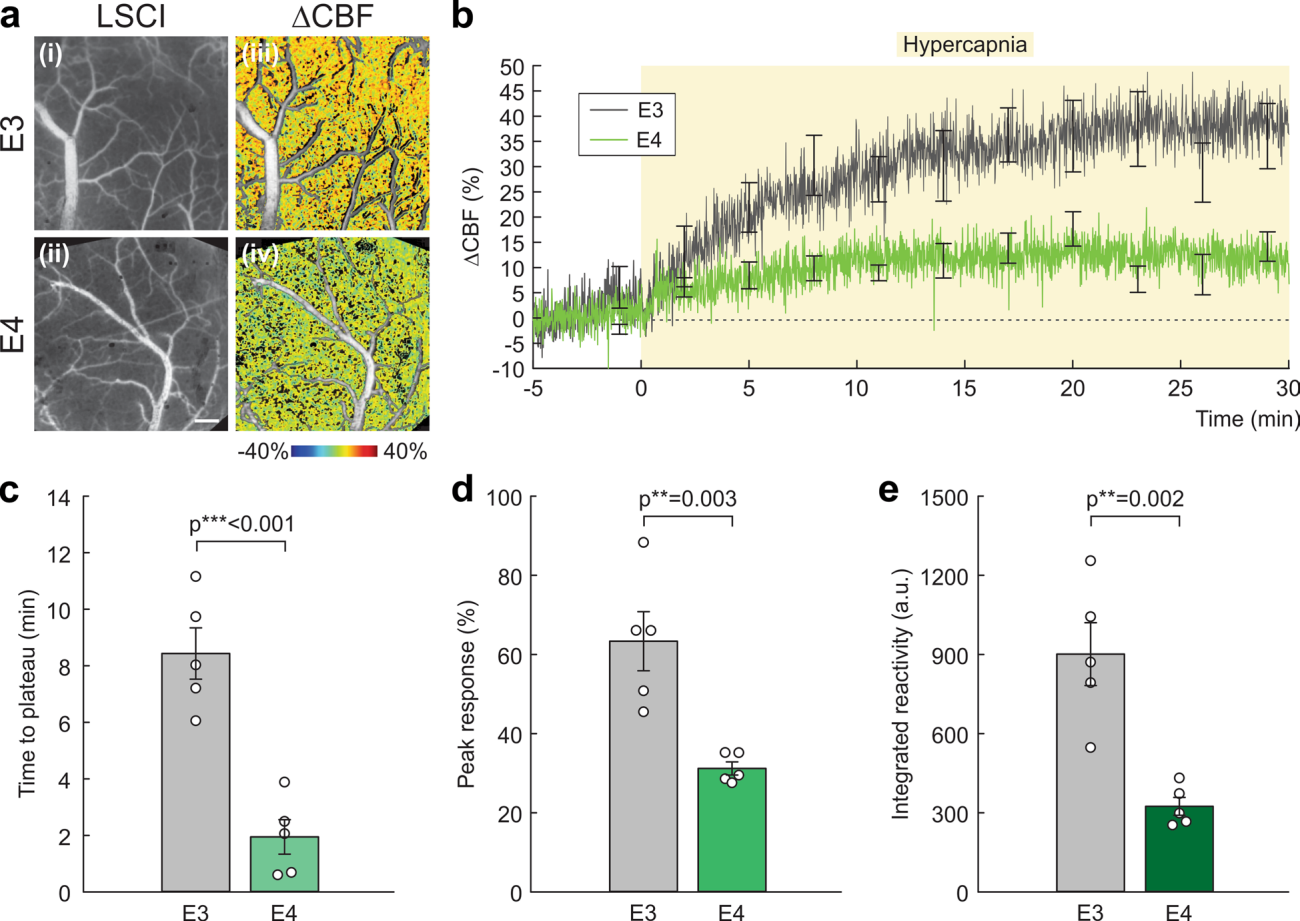
Dysfunctional cortical CBF response to a vasodilatory stimulus (hypercapnia) in E4 vs E3. **a** Raw laser speckle contrast imaging (LSCI) (**i**, **ii**) and their ratio images (ΔCBF, **iii**, **iv**) to illustrate spatial CBF response in the microvasculature to hypercapnia via inhalational 5% CO_2_ in E3 and E4 mice, which showed a substantial reduction in hypercapnia-induced ΔCBF increase in E4. Scale bar: 300 µm. **b** Time-lapse tracking of ΔCBF(%) in the cortex of E3 (gray) and E4 (green) mice (*n* = 6/group) from normocapnia (*t* ≤ 0min, 100% O_2_) to hypercapnia (*t* > 0 min, 5% CO_2_: 95% O_2_). Error bars are SEM at every 3 min. **c** Time to reach a plateau (*t*_P_; min ± SEM) for vessel dilation after hypercapnia in E3 and E4 mice, which was significantly shorter in E4, possibly due to reduced compliance requiring shorter time to reach their maximum blood volume capacity. **d** Peak ΔCBF response (ΔCBF_p_; % ± SEM) to hypercapnia in E3 and E4, which was significantly reduced in E4. **e**. Integrated reactivity (IR; a.u. ± SEM), the extent of vasodilation to hypercapnia. E4 responded significantly less than E3, which may be a sign of stiffness and limited vasodilatory capacity in the vasculature

To evaluate CVR as a measure of cerebral functionality (e.g., vasodilatory capacity) of E3 and E4 mice, we applied hypercapnia and recorded ΔCBF(%) in the microvasculature using LSCI (Fig. 4). Representative CBF images of E3 and E4 cortices and their ratio images of ΔCBF showed distinctively reduced spatial distribution of vasodilatory perfusion in E4 (Fig. 4a). Figure 4b presents the averaged in vivo tracking (*n* = 5/group) of ΔCBF(t) from normocapnia (*t* ≤ 0min) to hypercapnia (*t* > 0 min). To assess the differences, we quantified (1) time to reach a plateau (*t*_P_), (2) peak response (ΔCBF_p_), and (3) integrated reactivity (IR). Figure 4c shows that *t*_P_ in E4 (1.94 ± 0.61 min) was significantly shorter than that in E3 (8.41 ± 0.90 min; *P* < 0.001). This large difference may also reflect the limited vasodilatory capacity as collaterally indicated by reduced ΔCBF_p_ and IR (accumulative CVR response) in E4 vs E3 mice. The ΔCBF_p_ in E4 (31.18% ± 1.65%) was significantly lower than in E3 (63.35% ± 7.46%; *P* = 0.003) (Fig. 4d). The IR (a.u.) in E4 (324.28 ± 33.84) was even lower than that in E3 (901.66 ± 119.1; *P* = 0.002) (Fig. 4e).

**Fig. 5:**
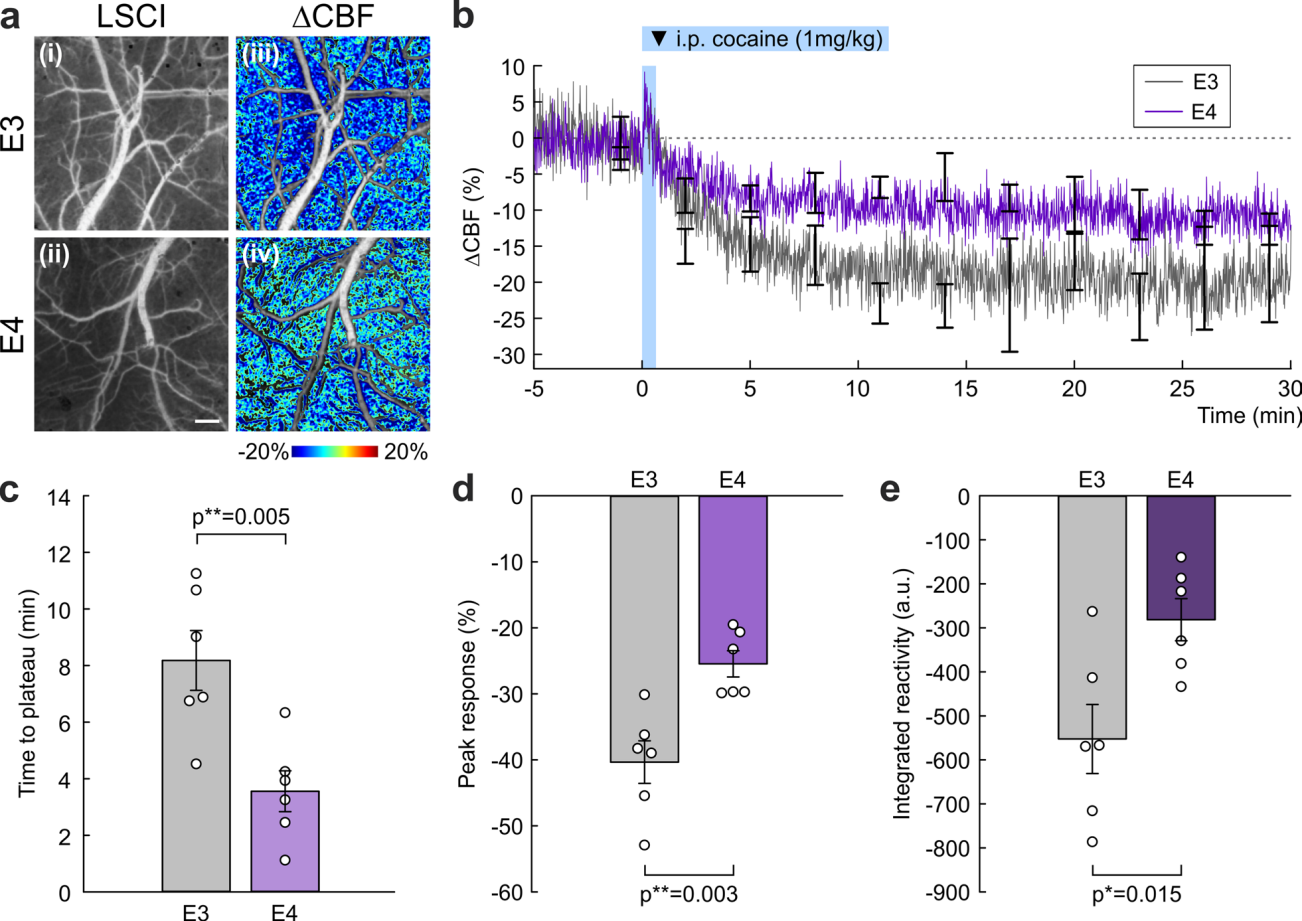
Dysfunctional cortical blood flow response to a vasoconstrictive stimulus (cocaine) in E4 mice. **a** LSCI images of spatial cortical CBF response to acute cocaine injection in E3 (**i**) and E4 (**ii**). The correlation masking between before and after cocaine periods was applied to the respective LSCI images (**iii, iv**). Scale bar: 300 µm. **b** Continuous in vivo tracking of ΔCBF in E3 and E4 mice (*n* = 6/group) before and after acute administration of cocaine (1 mg/kg, i.p.). Error bars are SEM at every 3 min. **c** Time to reach a plateau (*t*_P_; min ± SEM) after cocaine injection in E3 and E4. E4 took a significantly shorter time to constrict the vessels, which may be due to reduced vessel diameters and dynamic range to accommodate the vasoconstrictive stimulus. **d** Peak ΔCBF response (ΔCBF_p_; % ± SEM), i.e., the maximum decrease in CBF after cocaine injection. E4 showed a significantly reduced response. **e** Integrated reactivity (IR; a.u. ± SEM), the extent of vasoconstriction from cocaine. E4 responded significantly less than E3

In contrast to vasodilation, the vasoconstrictive capacity was examined through cocaine administration (1 mg/kg, i.v.). Figure 5a presents representative CBF images of E3 and E4 and their ratio images of ΔCBF to visualize their responses to cocaine. Figure 5b presents the averaged in vivo quantitative tracking (*n* = 6/group) of ΔCBF(t) from baseline to 30 min after cocaine injection at *t* = 0 min. The *t*_P_ in E4 (3.56 ± 0.72 min) was significantly shorter than that in E3 (8.18 ± 1.06 min; *P* = 0.005) (Fig. 5c). The ΔCBF_p_ in E4 (− 25.45% ± 1.99%) was less than that in E3 (− 40.34% ± 3.22%; *P* = 0.003) (Fig. 5d). The IR in E4 (− 281.41 ± 47.78) was lower than that in E3 (-552.57 ± 78.57; *P* = 0.015) (Fig. 5e). Taken together, both vasodilatory and vasoconstrictive CVRs are impaired in the cortical vascular network of E4 mice.

### AI-driven analysis of 36 vascular parameters for detecting E4-specific neurovascular deficits

**Fig. 6:**
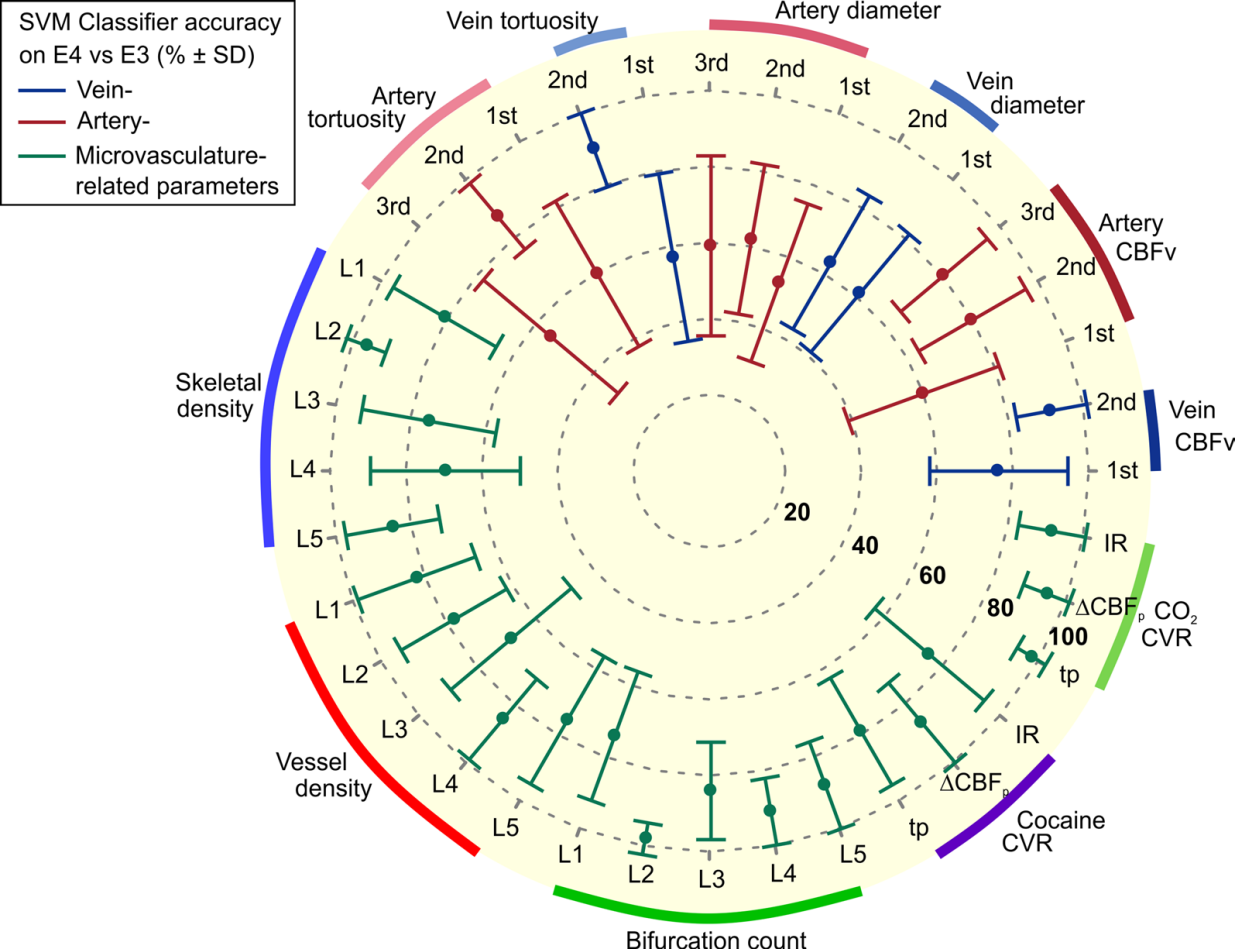
AI-driven systematic evaluation of the measured 36 vascular parameters for E3/E4 classification accuracy. The radar chart showing the classification accuracy score (% ± SD) of each vascular parameter in distinguishing E4 from E3 by machine-learning Support Vector Machine (SVM). Vein-, artery-, and microvasculature-related parameters are shown in blue, red, and green, respectively. The microvasculature-related parameters showed relatively higher accuracy scores (~ 84% on average). Also, higher-order venules showed more robust accuracy compared to 1st-order (pial) vessels. This AI-powered multi-parameter analysis suggests E4-specific neurovascular alterations in deeper cortical layers and smaller vessels, demonstrating the potential of these vascular biomarkers for enhanced, early detection of E4-related neurodegeneration

To systematically evaluate the most sensitive neurovascular parameters for E3 vs E4 detection, we built an AI-based classification framework—an SVM classifier. Figure 6 and Table S1 show the mean accuracy scores (%) with standard deviation as error bars and 95% confidence intervals of the 36 vascular parameters including 6 of vein-, 9 of artery-, and 21 of microvasculature-related features collected via the OCT and LSCI images. The microvasculature-related parameters showed higher accuracy scores (83.6% ± 2.0% on average) than other vessel types (66.1% ± 4.25% and 73.0% ± 6.1%, for vein- and artery-related parameters on average, respectively). Also, the parameters with accuracy higher than 90% included bifurcation count of L2 and L4, skeletal density of L2, *t*_P_, ΔCBF_p_, and IR of CO_2_ CVR, and 2nd order of venular in CBFv and tortuosity. This demonstrated the robustness of microvasculature and smaller veins/venules as well as vasoactive CVR as highly sensitive vascular metrics for E4-specific pathology detection. Overall, this analysis suggests that the E4-specific neurovascular alterations are mostly found in deeper cortical layers and smaller vessels.

To ensure that systemic physiology did not confound the classifier results, we performed a series of two-way repeated measures ANOVA on body temperature (°C) and respiration rate (breaths/min) recorded throughout the three imaging sessions (OCT, LSCI-cocaine, and LSCI-CO_2_) with Group (E3 vs E4) and Time (*t* = 1–35 min) as factors. The results revealed no significant effects of Group or Time and no significant Group × Time interaction for both parameters in any imaging session, confirming that systemic physiology was unlikely to confound the observed group differences in the vascular parameters. Detailed ANOVA results are provided in Table S2.

## Discussion

Our study provides a comprehensive assessment of structural and functional alterations of the cerebrovasculature associated with the *APOE4* gene via the combined use of in vivo optical imaging modalities and AI-assisted analyses for improved precision and objectivity. We found that resting-state CBFv and diameters of both venules and arterioles were reduced in 12-month-old E4 mice and that their CVRs to both vasoconstrictive and vasodilatory stimuli were impaired compared to those of E3 mice. In vivo*,* 3D microvascular imaging of different cortical layers revealed that the density and bifurcation counts of microvessels were significantly decreased in all layers of the E4 mice, especially in L2, suggestive of impaired angiogenesis and poor microcirculation that may disrupt neurovascular coupling. Additionally, a machine-learning-based SVM classifier enabled us to identify the most sensitive vascular biomarkers for detecting E4-specific impairment, which were mostly related to venules, layer 2 microvasculature, and hemodynamic response to vasoactive stimuli. Altogether, these data implicate significant E4-driven degradation in vascular morphology and vasoactivity prior to the onset of neurodegeneration. Given the crucial roles of E4 in multiple pathways that collectively affect cognitive function as well as the high risk it poses for AD/ADRD, we expect our findings to provide valuable insights into its role in vascular impairments and enhance early detection of AD/ADRD through the identified vascular biomarkers with high discriminative power.

In the past years, many transgenic murine models have been developed to elucidate the molecular and cellular mechanisms underlying the cause of AD and to test therapeutic strategies prior to clinical trials. Of them, several monogenic *APP* or bigenic *APP*/*PSEN1* models have been shown to exhibit vascular defects such as microbleeds, hypoperfusion, and arterial narrowing [47–49], which are often attributed to deposition of Aβ on the vasculature. For example, one of the most used *APP*/*PSEN* models, 5 × FAD, exhibits retinal vascular defects such as arterial narrowing and decreased microvascular in the retinal tissues [50, 51]. As opposed to these amyloidosis-driven AD lines, the E4-KI model used in this study revealed cerebrovascular abnormalities in the absence of amyloid or tau pathology. These findings suggest that E4 may independently drive vascular dysfunction, potentially contributing to increased AD/ADRD risk.

As opposed to conflicting results of CBF levels in E4-carriers measured using PET [52, 53] or blood-oxygenation-level-dependent (BOLD)-MRI [54], we demonstrated decreased venular and arteriolar CBFv as well as diameters in the SSC of E4 compared to that of E3 using µODT. However, a previous study using TPM did not find significant reductions in basal red blood cell (RBC) flux nor diameters of venules, arterioles, and capillaries in the neocortex of *APOE4*-targeted-replacement (TR) mice relative to that of E3-TR [21]. Instead, they observed reduced vascular density in the cortices of E4-TR via CD31 histological staining and reduced cortical CBF in resting state using arterial-spin-labeling -MRI. The authors concluded that the reduced CBF likely stemmed from decreased vascular density rather than flow changes in individual vessels. The discrepancy from our results may be attributed to methodological differences in CBF measures: TPM requires the use of an external fluorescent marker and repeated line scans in a limited FOV to estimate RBC speed (thus is subject to the vessel/flow selected), whereas OCT detects intrinsic Doppler shifts from moving RBCs without a tracker, allowing for quantitative CBFv imaging of multiple vessels over a much larger FOV. Also, presenting the vascular profile without distinguishing branch orders may blunt differences between groups as there is a positive correlation between blood flow velocity and diameter [55]. Hence, our OCT results offer new evidence on the pathological effect of E4 on individual vessels on an expanded FOV.

Abnormal vascular tortuosity is increasingly being recognized in many vasculopathies such as atherosclerosis [56] and stroke [57]. Using µOCA, we documented increased venular and arterial tortuosity in the cortex of E4 compared to that of E3—a feature observed in AD patients through retinal OCTA [58]. While the non-invasiveness of retinal imaging makes it accessible to clinical settings, it remains uncertain whether retinal changes mirror cerebral abnormalities or whether they reflect localized retinal impairments from systemic vascular risk factors [59]. Recently, a study characterized the ocular phenotype of E4-KI mice and reported retinal tortuosity and thinning along with functional deficits [60]. The authors attributed these deficits to pericyte impairment [61], neuroinflammation [62], and synaptic dysfunction from the downregulated synaptogenesis and glutaminergic receptor signaling in E4 [60], which may have effects on the cerebral circulation as well. Our findings of tortuous vessels in the SSC of E4 mice establish a direct parallel with these retinal observations, thereby highlighting the possibility of retinal tortuosity as a surrogate biomarker to monitor AD/ADRD progression [63]. The functional implications of increased cortical tortuosity in the SSC and their generalizability to other cortical brain regions remain an interesting topic for our ongoing studies.

Microvasculature has conventionally been studied using post-mortem histological staining with vessel-specific markers [64–66]. While this traditional method can visualize microvasculature in deeper tissues that are often inaccessible to in vivo imaging, it is limited by the cross-sectional nature of sampling, which may over- or under-estimate vascular density. Alternatively, optical imaging modalities such as ultrasound localization microscopy, photoacoustic tomography, TPM, and OCT are increasingly used in transgenic rodent models [67–69]. In addition to the previously stated merits of our OCT setup, our approach provided a more accurate estimation of vascular densities in a layer-dependent manner based on 3D-segmented vessels, which has remained a technical challenge, especially in vivo. Although a recent study using 3-photon-excitation fluorescence/third-harmonic-generation microscopy (3PM/THG) rendered the cortex of APOE4-TR up to 1000 µm depth in 3D [21], the reported data did not include quantitative in vivo vascular density measures. However, it should be noted that due to the axial resolution of our OCT system (~ 2.5 µm), precise evaluation of the smallest capillaries may be limited. Instead, our techniques enabled simultaneous imaging of microvascular architecture (µOCA) and complementary flow profile (µODT) over a wider FOV than 3PM/THG (0.2 × 0.2 mm^2^). This broader spatial coverage with direct comparison of vascular structure and flow allows for systemic, layer-dependent analysis of heterogenous cerebrovasculature. Altogether, our 3D reconstruction of microvasculature and quantification of vascular parameters across different layers offer an advanced tool for studying E4-driven cerebrovascular impairments in the deeper cortex.

The cerebral cortex consists of distinct layers with unique neuron subtypes that interconnect across the layers [70, 71]. The cortical circuitry of these layer-specific neurons and their roles in sensory processing have been extensively studied using in vitro IHC and electrophysiological recordings in brain slices, as well as in vivo rodents [72–75]. Recent evidence suggests that in an event of sensory stimuli, L2/3 pyramidal neurons evoke and amplify excitatory input to L5 pyramidal neurons [76], which then influence subcortical structures related to action [71]. Given the critical role of L2/3 neurons within the cortical circuitry for sensory processing, our results on the significant reduction in vascular skeletal density and bifurcation counts in L2 may signify pathological implications for neuronal excitability due to insufficient cerebral perfusion, contributing to sensory and cognitive deficits in AD/ADRD [77]. In studies from post-mortem brains of AD patients [78–80] and in transgenic AD rodents [81], early neuropathological changes appear to emerge in the superficial layers (L2-4) before progressing to deeper layers (L5-6). Especially, L2 and L3 of the sensory cortices exhibit more pronounced Aβ accumulation, tauopathy, and gliosis [82, 83], highlighting layer-dependent alterations that occur during the initiation/propagation of AD. These cellular changes align with our findings of selective vulnerability in superficial cortical layers, despite the systemic, vascular impairment observed across all cortical layers. However, it is important to note that this layer-specific pattern should be interpreted with caution, as it may not generalize to other cortical regions and depth-dependent signal limitations in deeper cortical layers could not be ruled out.

Physiologically, angiogenic growth factors such as vascular endothelial growth factor (VEGF) are reduced in AD patients with their levels correlated to the severity of cognitive impairment [84], and treatment with VEGF was reported to reverse E4-induced pathologies in humanized-E4 mice [85]. Given that a decline in these growth factors typically impairs angiogenic response to prevent hypoxia [86], this deficit may explain the observed reductions in CBFv, vascular density, and bifurcation counts in the cortex of E4. Overall, our data highlight vascular degradation possibly fueled by impaired angiogenic capabilities, suggesting potential therapeutic targets to mitigate the pathological effects of E4.

CVR serves as an indicator of vascular health and homeostatic ability to regulate blood supply in response to changing metabolic demands and physiological conditions [87]. Especially, the sensitivity to CO_2_ is crucial because CO_2_ signaling plays a complex role in mediating neurovascular coupling [88] and regulating CBF [89]. Hence, the decreased vasodilatory CVR in E4 suggests a reduced capacity to address neural insults, possibly amplifying neurodegenerative processes. Our CO_2_-CVR data are consistent with previous clinical studies, which used BOLD-fMRI [90]/transcranial Doppler ultrasonography [91]. Our study uniquely shows dynamic features using LSCI with detailed metrics—the delay (*t*_p_), amplitude (ΔCBF_p_), and IR of CBF changes to hypercapnia—in E4 mice.

Vasoconstrictive ability in response to elevated pressure or other stimuli is equally critical for maintaining CBF autoregulation and adapting to changing metabolic needs [92]. While measuring vasoconstrictive capacity has been challenging due to the risks associated with inducing ischemia in clinical subjects, we utilized a rodent model to demonstrate that vasoconstrictive CVR is significantly impaired in E4 compared to E3, which may be due to reduced vessel diameters and diminished dynamic range/compliance to accommodate the vasoconstrictive stimulus. These impairments in vasodilation and vasoconstriction may be attributed to E4-driven dysfunction in endothelial cells including oxidative damage and neuroinflammation [93] as the endothelial cells modulate vascular tone by releasing various vasoactive factors [94]. An in vitro BBB study of E4-KI astrocytes showed impaired endothelial cell tight junctions and compromised barrier integrity [95]. A subsequent in vivo study revealed that removing E4 from astrocytes prevented the observed endothelial dysfunction [96], suggesting a possible connection between the impaired vascular functionality and E4’s pathological effects on endothelial cells. Overall, vascular reactivity dysfunction may contribute to homeostatic dysregulation and neurological complications commonly seen in AD/ADRD patients.

Lastly, we employed an SVM classifier to evaluate the classification accuracy of individual vascular parameters and found that microvasculature-related parameters are particularly sensitive for early detection of E4-related pathology. Previously, machine-learning-based SVM has been applied in the classification of AD patients from cognitively normal individuals using MRI-based structural changes such as cortical thickness, tissue densities, and brain atrophy (summarized by Rathore et al. [97]). To our knowledge, this is the first study that applies SVM to systematically assess the predictive value of vascular morphological and functional parameters in middle-aged E4-KI mice as potential biomarkers for early detection of neurovascular impairment.

While our study provides extensive evidence on the pathological effect of E4 on the cerebrovasculature, several limitations should be acknowledged. Although our OCT system provides a high-resolution image at up to ~ 1.4 mm depth, there is still a constraint that only cortical layers can be captured. Given that there are certain brain regions such as the hippocampus that are known to be severely damaged in AD [98], further investigations into deeper brain regions would further enhance our understanding of the effect of E4. It is also important to note that LSCI captures projected blood flow changes from superficial layers (~ 1 mm), and thus layer-dependent dynamic differences could not be assessed for CVR. We did not apply a Bonferroni correction given that it can be overly conservative for small numbers of correlated comparisons so some of the significant findings would not survive correction for multiple comparisons and need to be interpreted cautiously [99]. Larger datasets to train SVM classifiers that include clinical data will be needed to assess the generalizability of this machine learning model for early detection of vascular pathology in individuals at risk for AD/ADRD. Nevertheless, our SVM classifier demonstrated robust classification accuracy for certain parameters, suggesting that they hold strong discriminatory power and potential as biomarkers for AD/ADRD in E4-carriers. Additionally, the cortical layers here were divided by segments with a fixed-depth interval of 100 μm/layer, which may not precisely correspond to the cytoarchitectural layers of the mouse cortex. Lastly, it should be noted that only female mice were tested in this study because studies have shown that the effects of *APOE4* on AD risk are strongly sex-dependent, with the association and pathology being more pronounced in women than in men [100–103]. Future work is needed to determine whether comparable vascular alterations are also present in male *APOE4*-KI mice.

## Conclusion

Our study revealed the pathological contributions of *APOE4* on the structure and function of cortical vasculature and demonstrated sensitivity of vessel- and layer-specific vascular parameters as strong diagnostic biomarkers, especially those related to venules and microvasculature, using (1) ultrahigh-resolution OCT to precisely track CBFv, diameters, and tortuosity of individual vessels, (2) 3D imaging of CBFv networks over a wide FOV to assess layer-dependent vascular density and bifurcation counts, (3) LCSI to capture dynamic changes in CBF elicited by both vasodilatory and vasoconstrictive stimuli, and (4) machine-learning-based SVM classifier to evaluate the E3/4 classification accuracy of these parameters. These vascular pathological alterations, especially impaired angiogenesis of cortical L2 and microvascular reactivity, emphasize the selective vulnerability of the cortical microvasculature, which may contribute to neuronal dysfunction and neurodegeneration in E4-carriers. Accordingly, alternative therapeutic approaches to address cognitive impairment could be attempted, such as reducing E4 expression by targeting its receptors, gene-editing E4 with the protective E2 variant, or directly ameliorating cerebrovascular dysfunction [104]. Overall, we believe that these results significantly enhance our understanding of E4’s role in cerebrovascular dysfunction and suggest the potential for mitigating its risks on neurodegeneration [105].

## Supplementary Information

Additional file 1. **Table S1** Mean classification accuracies of 36 vascular parameters with standard deviation and lower/upper bound confidence intervals. **Table S2** Results of two-way repeated measures ANOVA assessing systemic physiology data during three imaging sessions.

## Data Availability

Data sets generated during the current study are available from the corresponding author upon reasonable request.

## Abbreviations

AD: Alzheimer’s disease
ADRD: AD-related dementias
APOE: Apolipoprotein E
KI: Knock-in
Aβ: Amyloid-beta
BBB: Blood–brain barrier
IHC: Immunohistochemistry
fMRI: Functional magnetic resonance imaging
PET: Positron emission tomography
TPM: Two-photon microscopy
FOV: Field-of-view
SVM: Support vector machine
OCT: Optical coherence tomography
OCA: Optical coherence angiography
CBFv: Cerebral blood flow velocity
ODT: Optical doppler tomography
SSC: Somatosensory cortex
CVR: Cerebrovascular reactivity
LSCI: Laser speckle contrast imaging
RBF: Radial basis function
SEM: Standard error of the mean
RBC: Red blood cell
MIP: Maximum-intensity-projection
L: Layer
*t*_p_: Time to reach a plateau
ΔCBF_p_: Peak reseponse
IR: Integrated reactivity
BOLD: Blood-oxygenation-level-dependent
3PM/THG: 3-Photon-excitation fluorescence/third-harmonic-generation microscopy
VEGF: Vascular endothelial growth factor
