# Supplementary material for: Cortical layer multi-parameter analysis of neurovascular impairments in AD/ADRD rodent model with in vivo optical imaging
Source: Transl Neurodegener. 2025 Dec 22;14:70. doi: 10.1186/s40035-025-00530-4 (PMC12720472; doi:10.1186/s40035-025-00530-4)
Supplement: Supplementary file 1 — Additional file 1. Table S1 Mean classification accuracies of 36 vascular parameters with standard deviation and lower/upper bound confidence intervals. Table S2 Results of two-way repeated measures ANOVA assessing systemic physiology data during three imaging sessions. [file 40035_2025_530_MOESM1_ESM.docx]

**Supplemental Materials**

**Cortical layer multi-parameter analysis of neurovascular impairments in AD/ADRD rodent model with *in vivo* optical imaging**

Hyomin Jeong^1^, Jiaxiang Ren^2^, Wensheng Cheng^2^, Nora D Volkow^3^, Haibin Ling^2^, Donghui Zhu^1^, Congwu Du^1^, Yingtian Pan^1*^

^1^Department of Biomedical Engineering, Stony Brook University, Stony Brook, NY 11794, United States of America;

^2^Department of Computer Science, Stony Brook University, Stony Brook, NY 11794, United States of America;

^3^National Institute on Alcohol Abuse and Alcoholism, National Institutes of Health, Bethesda, MD 20857, United States of America

*During the review process, please address all correspondences to:

Yingtian Pan, PhD

Professor

Department of Biomedical Engineering

State University of New York at Stony Brook

Bioengineering Bldg, Rm. G17

Stony Brook, NY 11794-5281, United States

Tel: (631) 632-1519 (Office)

(631) 632-1750 (Lab)

Email: [yingtian.pan@stonybrook.edu](mailto:yingtian.pan@stonybrook.edu)

**Table S1: Mean classification accuracies of 36 vascular parameters with standard deviation and lower/upper bound confidence intervals.**

| **Vascular parameter** | **Mean accuracy (%)** | **Standard deviation** | **Lower bound CI** | **Upper bound CI** |
| --- | --- | --- | --- | --- |
| Vein_CBFv_1st_order | 75.71 | 18.17 | 30.77 | 100.00 |
| Vein_CBFv_2nd_order | 90.87 | 9.38 | 69.23 | 100.00 |
| Artery_CBFv_1st_order | 59.54 | 21.13 | 15.38 | 92.31 |
| Artery_CBFv_2nd_order | 79.37 | 16.28 | 38.46 | 100.00 |
| Artery_CBFv_3rd_order | 80.03 | 14.87 | 41.67 | 100.00 |
| Vein_Diameter_1st_order | 61.02 | 20.18 | 15.38 | 92.31 |
| Vein_Diameter_2nd_order | 63.25 | 20.17 | 15.38 | 92.31 |
| Artery_Diameter_1st_order | 52.67 | 22.02 | 0.00 | 84.62 |
| Artery_Diameter_2nd_order | 61.82 | 19.93 | 15.38 | 92.31 |
| Artery_Diameter_3rd_order | 59.19 | 23.65 | 0.00 | 90.00 |
| Vein_Tortuosity_1st_order | 56.95 | 22.31 | 15.38 | 92.31 |
| Vein_Tortuosity_2nd_order | 90.16 | 10.71 | 61.54 | 100.00 |
| Artery_Tortuosity_1st_order | 59.86 | 22.02 | 7.69 | 92.31 |
| Artery_Tortuosity_2nd_order | 87.36 | 11.28 | 61.54 | 100.00 |
| Artery_Tortuosity_3rd_order | 55.00 | 23.15 | 0.00 | 91.67 |
| Skeletaldensity_L1 | 80.87 | 15.65 | 41.67 | 100.00 |
| Skeletaldensity_L2 | 96.39 | 5.41 | 83.33 | 100.00 |
| Skeletaldensity_L3 | 75.28 | 17.88 | 33.33 | 100.00 |
| Skeletaldensity_L4 | 69.84 | 19.71 | 25.00 | 100.00 |
| Skeletaldensity_L5 | 84.98 | 12.50 | 50.00 | 100.00 |
| Vesseldensity_L1 | 82.33 | 16.47 | 41.67 | 100.00 |
| Vesseldensity_L2 | 78.04 | 16.05 | 41.67 | 100.00 |
| Vesseldensity_L3 | 68.61 | 20.77 | 24.79 | 100.00 |
| Vesseldensity_L4 | 85.11 | 13.81 | 50.00 | 100.00 |
| Vesseldensity_L5 | 75.58 | 19.35 | 25.00 | 100.00 |
| Bifurcationcount_L1 | 74.10 | 18.01 | 25.00 | 100.00 |
| Bifurcationcount_L2 | 98.05 | 4.21 | 83.33 | 100.00 |
| Bifurcationcount_L3 | 84.02 | 12.82 | 50.00 | 100.00 |
| Bifurcationcount_L4 | 90.84 | 8.89 | 75.00 | 100.00 |
| Bifurcationcount_L5 | 87.87 | 12.15 | 58.33 | 100.00 |
| Cocaine_CVR_tP | 79.00 | 16.24 | 41.67 | 100.00 |
| Cocaine_CVR_ΔCBFp | 86.32 | 13.68 | 50.00 | 100.00 |
| Cocaine_CVR_IR | 74.99 | 18.84 | 25.00 | 100.00 |
| CO_2__CVR_tP | 97.87 | 4.92 | 80.00 | 100.00 |
| CO_2__CVR_ΔCBFp | 94.51 | 7.11 | 80.00 | 100.00 |
| CO_2__CVR_IR | 91.31 | 9.22 | 70.00 | 100.00 |

Vein-related parameters are marked in blue, artery in red, and microvasculature in green.

**Table S2: Results of two-way repeated measures ANOVA assessing systemic physiology data during three imaging sessions**

| **Imaging session** | **Physiological variable** | **Factor** | **df** | **F** | **p-value** |
| --- | --- | --- | --- | --- | --- |
| **OCT** | **Body temperature** | Group | 1, 350 | 1.317 | 0.252 |
|  |  | Time | 34, 350 | 0.476 | 0.995 |
|  |  | Group x Time | 34, 350 | 0.340 | 1.000 |
|  | **Respiration** | Group | 1, 350 | 0.709 | 0.400 |
|  |  | Time | 34, 350 | 0.698 | 0.899 |
|  |  | Group x Time | 34, 350 | 0.275 | 1.000 |
| **LSCI-cocaine** | **Body temperature** | Group | 1, 280 | 0.942 | 0.333 |
|  |  | Time | 34, 280 | 0.203 | 1.000 |
|  |  | Group x Time | 34, 280 | 0.328 | 1.000 |
|  | **Respiration** | Group | 1, 280 | 0.079 | 0.779 |
|  |  | Time | 34, 280 | 0.304 | 1.000 |
|  |  | Group x Time | 34, 280 | 0.429 | 0.998 |
| **LSCI-CO_2_** | **Body temperature** | Group | 1, 280 | 0.237 | 0.627 |
|  |  | Time | 34, 280 | 0.289 | 1.000 |
|  |  | Group x Time | 34, 280 | 0.263 | 1.000 |
|  | **Respiration** | Group | 1, 280 | 1.512 | 0.220 |
|  |  | Time | 34, 280 | 0.158 | 1.000 |
|  |  | Group x Time | 34, 280 | 0.266 | 1.000 |
